# Effect of Autologous Skin Cell Suspensions Versus Standard Treatment on Re-Epithelialization in Burn Injuries: A Meta-Analysis of RCTs

**DOI:** 10.3390/medicina61030529

**Published:** 2025-03-18

**Authors:** Faisal M. Obeid

**Affiliations:** Anesthesia and Surgery Department, Faculty of Medicine, Imam Mohammad Ibn Saud Islamic University (IMSIU), Riyadh 11564, Saudi Arabia; fmobeid@imamu.edu.sa

**Keywords:** autologous skin cell suspension, burn injuries, re-epithelialization, partial-thickness burns, scar quality

## Abstract

*Background and Objectives*: Burn injuries, particularly partial-thickness burns, often require advanced therapies to improve re-epithelialization and scar quality. This study aims to evaluate the efficacy of autologous skin cell suspensions, such as Recell, compared to standard treatments in promoting faster and better-quality skin healing. Our goal is to provide evidence-based conclusions on the effectiveness of these regenerative approaches in burn treatment. *Materials and Methods*: During our comprehensive investigation, we systematically examined several databases for the period to November 2024, including PubMed, Scopus, Web of Science, and the Cochrane Central Register of Controlled Trials. Our primary objective was to assess the efficacy of autologous cell suspension in treatment for burn injuries. We employed the ROB2 method to assess the quality of evidence to ensure the validity of the conclusions derived from these studies. The gathered data were systematically organized in a standardized online format and analyzed with RevMan 5.4. *Results*: Our systematic literature search identified nine studies (n = 358 patients) evaluating the efficacy of autologous skin cell suspensions in promoting re-epithelialization in burn injuries. The meta-analysis revealed a statistically significant reduction in time to re-epithelialization in the autologous skin cell suspension group compared to the control group (MD = −1.71 days, 95% CI [−2.73, −0.70], *p* = 0.001), with moderate heterogeneity among the studies (I^2^ = 58%). However, no significant differences were found in secondary outcomes, including postoperative pain (SMD = −0.71, 95% CI [−2.42, 1.00], *p* = 0.42), POSAS scores (MD = −0.35, 95% CI [−2.12, 1.42], *p* = 0.69), Vancouver Scar Scale (MD = −0.76, 95% CI [−2.86, 1.33], *p* = 0.48), or the incidence of complete healing by the 4th week (RR = 0.98, 95% CI [0.94, 1.02], *p* = 0.24). Similarly, no significant differences were found in postoperative infection rates (RR = 0.85, 95% CI [0.28, 2.60], *p* = 0.78) or the need for further interventions (RR = 0.15, 95% CI [0.02, 1.16], *p* = 0.07). *Conclusions*: autologous skin cell suspension significantly reduces the time to re-epithelialization in burn injuries compared to standard treatments. However, no significant differences were found in secondary outcomes, such as postoperative pain, scar quality (POSAS, Vancouver Scar Scale), complete healing rates, infection rates, or the need for additional interventions. While autologous skin cell suspension shows promise in accelerating re-epithelialization, it does not provide significant advantages over conventional methods in other clinical aspects. The results underscore the need for further research with larger, more robust studies to assess the long-term benefits of autologous skin cell suspension in burns carefully.

## 1. Introduction

Burn injuries remain a significant global public health issue, impacting over 11 million individuals each year [1]. Age, profession, and socioeconomic factors affect both the occurrence of burn injuries and the mortality risk associated with burns [2]. The risk of fatal burns and burn injuries is highest in low-development countries due to outdated infrastructure, inadequate safety regulations, lack of smoke detectors, and faulty electrical systems, among other factors [3,4]. Re-epithelialization commences within 24 h post-injury [4]. A delayed time to re-epithelialization (TTRE) exceeding 21 days correlates with a markedly heightened risk of hypertrophic scar formation and unfavorable cosmetic results [5,6]. Consequently, contemporary burn wound management approaches prioritize reductions in total thermal injury, the minimization of scar formation, and the attainment of optimal cosmetic outcomes.

The treatment strategy for burn patients is dictated by the injury’s severity, with minor superficial burns typically treated on an outpatient basis using standard dressings. In contrast, more profound and extensive burns frequently necessitate hospitalization for thorough care and intervention [7]. Wound healing remains challenging, as contemporary medicine does not consistently offer effective remedies. Surgical interventions seek to accelerate the healing process while addressing essential issues, such as reducing immediate bleeding risks and preventing infections and their related complications [8]. Both meshed and non-meshed skin grafts are considered the gold standard and have demonstrated efficacy and simplicity over the years. The results unequivocally demonstrate that they expedite burn healing, yield favorable esthetic outcomes, and avert contractures, thereby establishing them as the most widely accepted approach for addressing epidermal loss [9,10].

Autologous cell harvesting, known as the autologous skin cell suspension system, has been explored in vitro and through animal studies as a novel approach for delivering epidermal cells to treat acute burns and wounds [11,12,13]. Introduced into clinical practice in 2005, autologous skin cell suspension was the first treatment of its kind. The development of ASCS marked a significant advancement in burn care, providing a faster and more efficient alternative to traditional skin grafting. It allows surgeons to quickly process a small split-thickness biopsy and deliver keratinocytes, melanocytes, fibroblasts, and Langerhans cells from the epidermal–dermal junction to the wound site. This technique has become a cornerstone in modern burn treatment, offering a more personalized and immediate solution to wound management. There are two primary methods for applying autologous non-cultured keratinocyte–melanocyte suspensions in burn treatment: most researchers use a syringe or spray technique to apply the suspension to prepared dermal skin, while others opt for injection directly into the dermis of the affected area [14,15]. The autologous skin cell suspension device was designed to reduce the need for extensive healthy skin grafting while ensuring complete wound closure. It enables the immediate application of autologous skin cells—without laboratory culturing—to support long-term wound healing efficiently, thereby improving clinical outcomes. The autologous skin cell suspension system generates an Autologous Skin Cell Suspension (ASCS) from an autograft measuring 0.006–0.008 inches thick at the point of care. Each 1 cm^2^ of donor skin produces 1 mL of suspension, capable of treating up to 80 cm^2^ of the wound area [16,17].

In this systematic review and meta-analysis, we aim to assess the efficacy of autologous cell suspension as a treatment for burn injuries.

## 2. Materials and Methods

### 2.1. Protocol and Registration

The Preferred Reporting Items for Systematic Reviews and Meta-Analyses (PRISMA) guidelines [18] and the instructions of the Cochrane Handbook for Systematic Reviews of Interventions [19] were followed in conducting this systematic review and meta-analysis. The study protocol was prospectively registered on the Open Science Framework (OSF) under the following DOI: https://doi.org/10.17605/OSF.IO/VEF2R, accessed on 12 February 2025.

### 2.2. Database Searching

We used the following search strategy—(ReCell OR (re-epithelialization suspension) OR (epidermal cell suspension) OR (skin cell suspension)) AND (burn or (partial-thickness burn) OR (full-thickness skin burn))—to scour four major databases (PubMed, Scopus, Web of Science, and Cochrane Central Register of Controlled Trials, or CENTRAL) for relevant published studies in our comprehensive literature review for the period to November 2024, with minor modifications made to accommodate the specific requirements of each database. Research articles published in English were the only ones taken into consideration during our search.

### 2.3. Eligibility Criteria

#### 2.3.1. Inclusion Criteria

All prospective comparative studies and randomized clinical trials (RCTs) were considered, including the following PICO studies:

- P: Patients with skin injuries, including thermal burns, flame burns, contact burns, injuries, electric arc injuries, or post-burn pigmentation issues.
- I: Autologous cell suspensions (ReCell), alone or with adjuncts like Biobrane or hydrosurgery.
- C: Standard treatment approaches (including classic skin grafting, Biobrane alone, surgical excision with dressings, or alternative delivery methods like the injection of cell suspensions.
- O: With a focus on efficacy outcomes.

#### 2.3.2. Exclusion Criteria

- No review;
- Letter to the editor;
- Abstract;
- Opinion;
- Study involving non-human subjects;
- Trials that compared abrocitinib to non-placebo controls;
- Trials that were not published in English.

### 2.4. Study Selection and Data Extraction

In order to eliminate repetitive data, the search results were imported into the EndNote program, specifically version 20 [20]. In order to find the papers that were appropriate for the study, two reviewers used the Rayyan program [21] to examine the titles and abstracts of the remaining papers. In the event that a third reviewer was required, the first two reviewers met to discuss and settle any differences in opinion. Articles were subjected to full-text screening once they fulfilled the inclusion criteria. We also looked through the chosen studies’ references for any possible further research to make sure we covered everything. Two independent reviewers used a pre-formatted Google Doc to record each study’s details, including their location, baseline characteristics, target population, publication year, and metrics for evaluating quality.

### 2.5. Outcomes

#### 2.5.1. Primary Outcomes

- Time recorded to re-epithelization.

#### 2.5.2. Secondary Outcomes

- Postoperative pain;
- Patient and Observer Scar Assessment Scale (POSAS) Overall opinion and total score [22];
- Vancouver Scar Scale Value [23];
- Incidence of complete healing in 4th week;
- Infection;
- Rate of patients requiring another intervention.

### 2.6. Risk of Bias Assessment

We used the revised Cochrane risk-of-bias tool for randomized trials (ROB 2) [24] to evaluate the risk of bias in the included clinical trials. The instrument is categorized into specific domains of bias, each addressing a unique facet of trial planning, execution, and reporting. A set of signaling questions is utilized within each domain to gather data concerning trial characteristics pertinent to bias risk. An algorithm generates a proposed evaluation of bias risk in each domain based on the responses to these inquiries. This evaluation may denote “Some concerns” or be categorized as having a “Low” or “High” risk of bias. Two independent evaluators assessed each domain individually, and a third evaluator’s judgment resolved any discrepancies.

### 2.7. Statistical Analysis

Review Manager software (RevMan v. 5.4) was used to conduct the meta-analysis, which ensured that there were at least two studies included and that data for the evaluated outcomes were available [25]. For continuous outcomes with a 95% confidence interval (CI), the effect size was calculated as the mean difference (MD). For different outcome reporting scales, the standardized mean difference (SMD) was used. In order to calculate the risk ratio (RR) and a 95% confidence interval (CI), the total number of patients and the number of events were combined for binary outcome data. A *p*-value less than 0.05 was chosen as the level of statistical significance. A mean and standard deviation were calculated from the interquartile range and median, respectively. Rather than modifying the baseline, the final values were extracted. The results were more conservatively estimated and generalizable because a random effect model (Inverse variance) was used instead of a fixed-effect model. To assess the existence and level of heterogeneity, we followed the procedures described in chapter nine of the Cochrane Handbook [19], specifically using the Chi-square and I-square tests. Results from the I-square test showed that heterogeneity, not chance, was responsible for the observed variation across studies, so we interpreted them as follows: 0% to 40% were deemed insignificant, 40% to 70% were deemed moderate, and 70% and above were deemed substantial. A Chi-square alpha value less than 0.1 was used to determine statistical significance for heterogeneity.

## 3. Results

### 3.1. Study Selection

**Figure 1:**
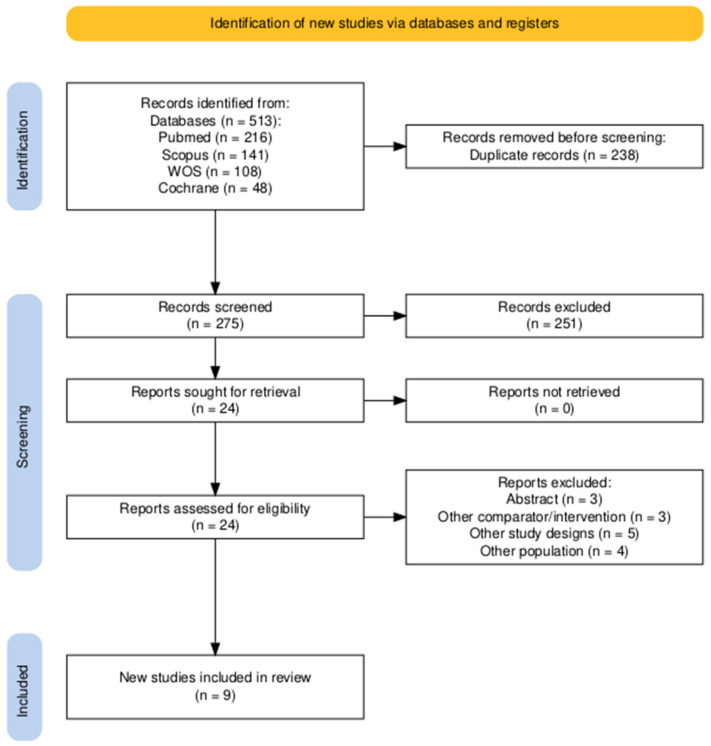
The PRISMA flow diagram of the selection process.

A systematic literature search yielded 513 potentially relevant records from PubMed, Cochrane Central, Scopus, and Web of Science databases. Endnote was used to eliminate duplicates, reducing the total to 275 records after removing 238 duplicates. Screening of titles and abstracts narrowed this to 24 articles that met our research criteria. After a full-text assessment, 15 records were excluded for specific reasons, leaving nine studies (Gravante 2007 [26], Sood 2015 [27], Wood 2012 [28], Guerid 2013 [29], Holmes 2018 [30], Holmes 2019 [31], Guo 2022 [32], Bairagi 2023 [33], and Iman 2013 [34]) for qualitative synthesis and meta-analysis. Additionally, the reference lists of these studies were manually checked to identify any relevant studies that might have been missed initially. Figure 1 illustrates the search process and the number of studies included and excluded.

### 3.2. Characteristics of the Studies

The systematic review encompassed nine studies published between 2007 and 2023, comprising diverse randomized controlled trials (RCTs) and prospective comparative studies across multiple geographical contexts, including Italy, India, Australia, Switzerland, the USA, China, and Iran. Study designs predominantly utilized RCT methodologies (n = 7) and two comparative studies, with populations ranging from pediatric to geriatric patients experiencing various burn injuries and wound healing scenarios. Total sample sizes varied considerably, with individual study populations ranging from 10 to 83 participants. Intervention protocols primarily centered on autologous cell harvesting techniques, including skin cell suspensions (ReCell), keratinocyte treatments, and regenerative epidermal suspensions. Follow-up periods demonstrated substantial variability, spanning from 1 week to 52 weeks. Comparative interventions included standard skin grafting techniques, Biobrane applications, surgical excision, and traditional wound care protocols. Primary outcome measures consistently focus on wound healing efficiency, donor site characteristics, pain reduction, functional outcomes, and esthetic wound appearance. Methodological heterogeneity was evident across studies, reflecting the evolving landscape of regenerative wound management strategies. Table 1 illustrates the summary of the trials involved, and Table 2 shows the baseline characteristics of included studies.

### 3.3. Risk of Bias and Quality Assessment

**Figure 2:**
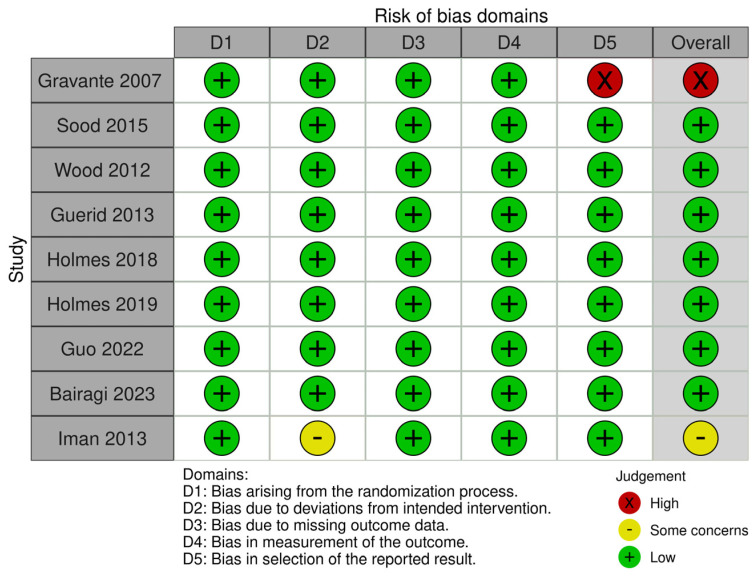
Risk of bias graph [26,27,28,29,30,31,32,33,34].

The evaluation of potential bias in the included randomized controlled trials (RCTs) was performed utilizing the ROB2 tool. Overall judgment of the included studies revealed that eight of the included clinical trials were judged as having a low risk of bias and one as having a high risk of bias. More details of the risk of biased judgments are shown in Figure 2.

### 3.4. Primary Outcomes

#### Time to Re-Epithelization

**Figure 3:**
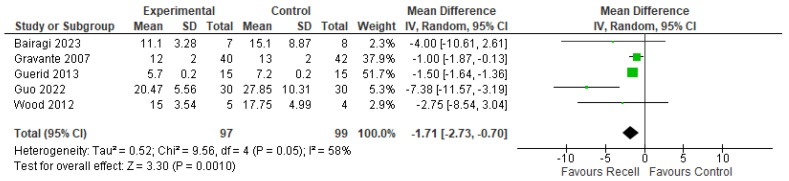
The time to re-epithelization [26,28,29,32,33].

Time to re-epithelization was recorded in five studies (Bairagi 2023, Gravante 2007, Guerid 2013, Guo 2022, and Wood 2012). A meta-analysis was conducted, and an effect estimate was calculated. The pooled-analysis resulted in a statistically significant lower time of re-epithelization in the autologous skin cell suspension group compared to the control group, favoring the use of autologous skin cell suspension (MD= −1.71 days, 95% CI [−2.73, −0.70], *p* = 0.001). Heterogeneity analysis showed moderate heterogeneity among studies ((*p* = 0.05); I^2^ = 58%). The forest plot of this outcome is illustrated in Figure 3.

### 3.5. Secondary Outcomes

#### 3.5.1. Postoperative Pain

**Figure 4:**
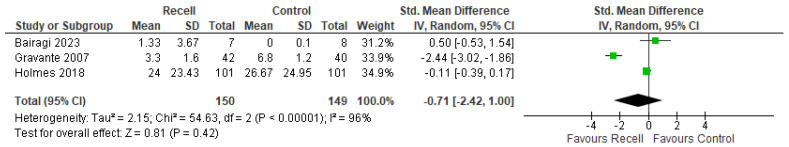
Operative pain [26,30,33].

Standardized mean differences were utilized due to different pain assessment tools. No statistically significant difference between groups was observed in the meta-analysis (SMD= −0.71, 95% CI [−2.42, 1.00]), as indicated by a *p*-value = 0.42. However, substantial heterogeneity was detected among studies ((*p* < 0.00001); I^2^ = 96%). The forest plot of pain outcome is illustrated in Figure 4.

#### 3.5.2. Patient and Observer Scar Assessment Scale (POSAS)

**Figure 5:**
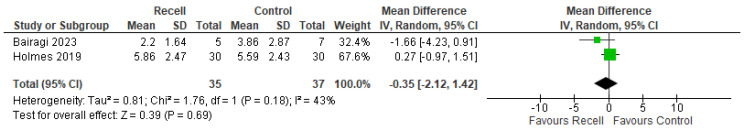
POSAS’s overall outcome [31,33].

**Figure 6:**
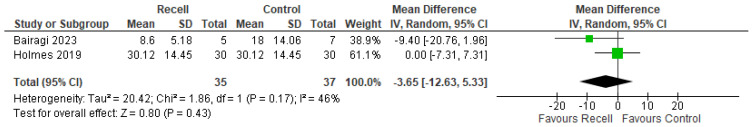
The POSAS total score [31,33].

The POSAS score is a comprehensive tool for evaluating scars, divided into an overall score (the average of individual parameters) and an opinion score (a single subjective assessment by either the patient or observer). POSAS was assessed in two included trials (Bairagi, 2023 and Holmes, 2019) [31,33]. No statistically significant differences were observed in POSAS overall opinion or POSAS total score ((MD = −0.35, 95% CI [−2.12, 1.42], *p* = 0.69) and (MD = −3.65, 95% CI [−12.63, 5.33], *p* = 0.43), respectively), with no significant heterogeneity detected (((*p* = 0.18); I^2^ = 43%) and ((*p* = 0.17); I^2^ = 46%), respectively), as shown in Figure 5 and Figure 6.

#### 3.5.3. Vancouver Scar Scale

The analysis showed no statistically significant differences between the autologous skin cell suspension group and the control group in terms of the Vancouver Scar Scale (MD = −0.76, 95% CI [−2.86, 1.33], *p* = 0.48), with substantial heterogeneity detected ((*p* < 0.00001); I^2^ = 96%). (Figure S1 in the Supplementary Materials.)

#### 3.5.4. Incidence of Complete Healing in the 4th Week

When comparing the autologous skin cell suspension group to the control group, no statistically significant differences were observed (RR = 0.98, 95% CI [0.94, 1.02], *p* = 0.24), with no heterogeneity detected ((*p* = 0.87); I^2^ = 0%), as shown in Figure S2 in the Supplementary Materials.

#### 3.5.5. Infection

The comparison of postoperative infection rates between the autologous skin cell suspension group and the control group showed no statistically significant difference (RR = 0.85, 95% CI [0.28, 2.60], *p* = 0.78), and no heterogeneity was detected ((*p* = 0.73); I^2^ = 0%), as shown in Figure S3 in the Supplementary Materials.

#### 3.5.6. Patients Requiring Another Intervention

Meta-analysis showed no difference between autologous skin cell suspension and the control group (RR = 0.15, 95% CI [0.02, 1.16], *p* = 0.07), without detecting heterogeneity ((*p* = 0.50); I^2^ = 0%) (Figure S4 in the Supplementary Materials).

## 4. Discussion

Burn wound management focuses on reducing thermal injury, minimizing scarring, and achieving optimal cosmetic outcomes, with the autologous skin cell suspension system providing a promising approach by harvesting and transplanting autologous skin cells to promote quicker healing and re-epithelialization [35]. Autologous skin cells aid in faster re-epithelialization by accelerating wound closure through immediate cell transplantation, stimulating tissue regeneration, and reducing the risk of hypertrophic scarring [36].

- **Main findings**

The key findings of this systematic review and meta-analysis suggest that autologous skin cell suspension offers significant benefits in reducing the time to re-epithelialization in burn injuries compared with standard care treatments. This indicates that autologous skin cell suspension can speed up healing, leading to quicker recovery in burn patients. However, despite this positive outcome, no clinically relevant difference in secondary outcomes could be measured concerning postoperative pain, scar quality (measured by POSAS and Vancouver Scar Scale), infection rates, or further interventions. This suggests that while autologous skin cell suspension may accelerate the initial phase of healing, it has little influence on the later stages of recovery or the quality of the healed skin.

In the case of postoperative pain, the meta-analysis did not show any significant difference between the autologous skin cell suspension and control groups. It indicates that autologous skin cell suspension does not have a distinct advantage in pain reduction, and the pain improvement may differ from that of conventional treatments. Moreover, during the evaluation of scar quality by POSAS and the Vancouver Scar Scale, no significant improvements were observed in the autologous skin cell suspension group when compared to control treatments. In all these scales, there was no difference, which points to the fact that though autologous skin cell suspension may help in faster healing, it does not significantly improve the esthetic or functional outcomes of the scar in the long term, such as in terms of elasticity, pigmentation, or patient satisfaction.

Incidence of complete healing by the fourth week, infection rates, and the need for additional interventions did not show any statistical significance among the groups. These findings indicate that autologous skin cell suspension is not necessarily more effective in preventing complications or reducing the need for further treatment than conventional methods. This fact underlines that while autologous skin cell suspension accelerates the initial healing phase, it does not provide any advantages concerning the success of the treatment in general, prevention of infection, or avoidance of secondary interventions.

It is noteworthy that marked heterogeneity was pointed out among the included studies. Much of this heterogeneity can be related to the significant variability in the application of standard care across trials. Although standard care was the comparator in all the studies, different approaches were adopted for standard treatment, like dressing types, pain management protocols, or strategies for wound care, across the other studies. These differences in comparator treatments contributed to the observed heterogeneity in outcomes, influencing the consistency of conclusions that can be derived across these studies.

- **Clinical implications**

The clinical implications are that these findings indicate the value of autologous skin cell suspension as an option for accelerating the re-epithelialization process in a burn injury, which reduces the time to closure versus standard care. This may enable faster recoveries and shorter lengths of hospital stay, which is important for patients and healthcare systems. However, given that secondary outcomes like postoperative pain, scar quality, and infection rates did not yield significant differences, clinicians must be cautious when assuming that autologous skin cell suspension would significantly improve in such respects. Another aspect that needs consideration is that no significant difference in further interventions could have resulted, meaning that it does not cut down on the risk of complications or re-treatments. Clinicians should consider autologous skin cell suspension within the framework of a multimodal treatment plan. Still, its use should be tempered with realistic expectations regarding the impact on long-term outcomes and scar management. Further research is needed to explore its full potential and optimize its integration into clinical practice.

- **Limitations**

Significantly, certain limitations must be acknowledged when analyzing the results. Initially, we were unable to obtain the results of Sood 2015 et al. due to their failure to report the units of variation. Secondly, studies documenting significant outcomes pertinent to treatment efficacy, including scar thickness, simplicity of dressing application, Observer Scar Assessment Scale (OSAS) [37], and Brisbane Burn Scar Impact Profile (BBSIP) [38], were inadequate for conducting a pooled analysis. Heterogeneity was observed in certain outcomes, likely attributable to variations in comparators, burn severity, extent of the affected area, materials utilized in the procedure, surgical techniques employed by the operating surgeons, and postoperative protocols. Nevertheless, these variations confer significant generalizability and external validity to the study. Furthermore, we were unable to perform subgroup analysis based on varying comparators due to the insufficient number of studies within each subgroup. Ultimately, we were unable to conduct publication bias or leave-one-out analysis to assess the robustness of the results due to the restricted number of studies.

- **Previous research**

Bairagi et al. [33] also performed a meta-analysis of a similar nature and came to the same conclusion: autologous skin cell suspensions (ASCS) may shorten the time to re-epithelialization in split-thickness skin graft donor site wounds in adults and partial-thickness burn wounds in children. They did, however, point out that there was not enough proof to definitively state that ASCS was useful for treating partial-thickness burns. They focused on a study by Hu et al. (2017), among several other factors that limited their analysis [39], which focused on donor-site wounds rather than burn injuries. Furthermore, the meta-analysis primarily relied on a single study for most outcomes; only five trials were included. In contrast, our systematic review and meta-analysis involved a more sophisticated analysis, including a larger set of trials (nine studies). This increased the robustness of our findings and allowed for a more comprehensive evaluation of ASCS efficacy in burn injury treatment.

## 5. Conclusions

In summary, while autologous skin cell suspension significantly reduces the time to re-epithelialization in burn injuries, the benefits of autologous skin cell suspension over standard care regarding secondary outcomes such as pain relief, scar quality, and long-term healing are limited. The results indicate that autologous skin cell suspension accelerates the initial healing phase but does not contribute much to the other important clinical features. These results would place autologous skin cell suspension as an option for improving early wound healing. However, further studies must confirm its long-term effects on scar formation, complication rates, and treatment outcomes.

## Figures and Tables

**Table 1:** A summary of the included studies.

Study ID	Design	Location	Population	Interv.	Comparator	Follow Up	Main Finding
Gravante 2007 [26]	RCT	Italy	Patients with deep partial-thickness burns	Autologous skin cell suspensions (ReCell) n = 42	Classic skin grafting in 40	6 M	ReCell demonstrated similar but slower functional outcomes compared to classic grafting, with reduced postoperative pain.
Sood 2015 [27]	Randomized prospective comparative study	India	Patients with a partial-thickness to deep partial-thickness burn of 4–25% of total body surface area	Autologous cell harvesting	(MSTSG)	52 weeks	Patients treated with ReCell had smaller donor site sizes and achieved comparable esthetic outcomes to those treated with meshed split-thickness skin grafting (MSTSG).
Wood 2012 [28]	Pilot RCT	Australia	Pediatric patients with partial-thickness scald injury	Autologous skin cell suspensions (ReCell) + Biobrane	G2: Biobrane alone, G3: standard treatment care (dressing)	6 M	The combination of ReCell and Biobrane resulted in faster healing, fewer dressing changes, reduced pain, and better scar outcomes compared to Biobrane alone in pediatric scald burns.
Guerid 2013 [29]	RCT	Switzerland	Patients aged between 18 and 80 years and presenting graft donor sites not exceeding 15% of TBSA	Keratinocytes suspended in autologous platelet concentrate (PC + K)	G2: Autologous platelet concentrate (PC), G3: Standard care	1 week	The use of autologous platelets with keratinocyte suspension showed promising results in enhancing wound healing and reducing donor site pain.
Holmes 2018 [30]	Within-body RCT	USA	Adult patients with an acute, deep partial-thickness thermal burn from 1% to 20% TBSA that required autografting	Autologous cell harvesting (Recell)	(MSTSG)	12 M	ReCell provided comparable healing to STSG in acute burn injuries, with significantly smaller donor site sizes, reduced pain, and improved cosmetic outcomes.
Holmes 2019 [31]	Within-body RCT	USA	Patients aged 5 years or older with mixed-depth burn injuries	Autologous cell harvesting (Recell)	(MSTSG)	12 M	ReCell reduced mean donor skin use by 32% while maintaining wound healing outcomes comparable to standard treatments in mixed burn injuries.
Guo 2022 [32]	Prospective comparative study	China	Patients with acute electric arc injuries	Hydrosurgery + RECELL	Surgical excision + dressing (standard care)	NR	Patients treated with hydrological debridement and autologous skin cell suspension showed faster healing and improved scar appearance compared to traditional methods in electrical burns.
Bairagi 2023 [33]	RCT	Australia	Children (age 16 years; 5% TBSA; 48 h of medium-size partial-thickness burns)	Regenerative epidermal suspension (Recell) + Biobrane	G2: Biobrane, G3: Standard dressing	12 M	The combination of regenerative epidermal suspension and Biobrane in partial-thickness burns led to lower pain, fewer infections, no sepsis, no need for skin grafts, and minimal impact on health-related quality of life.
Iman 2013 [34]	Prospective comparative study	Iran	Patients with post-burn hypo/depigmentation	Epidermal cell suspension spray	Epidermal cell suspension injection	9–15 months	There was no significant difference in outcomes between cell spray and intradermal injection methods, and both were unsatisfactory for patients and physicians clinically.

MSTSG: meshed split-thickness skin graft; STSG: split-thickness skin graft; PC + K: autologous platelet concentrate + keratocytes.

**Table 2:** The baseline characteristics of patients in the included studies.

Study ID	Arms	N	Age (Years), Mean (SD)	Male, n	Skin Lesion Area, cm^2^, Mean (SD)
Gravante 2007 [26]	Recell	42	49 (9)	24 (57.14)	186 (96)
	Skin grafting	40	53 (10)	26 (40)	180 (100)
Sood 2015 [27]	Autologous cell harvesting	10	44.4 (11.55)	9 (90)	NR
	MSTSG				
Wood 2012 [28]	Recell + Biobrane alone	5	1.32 (0.55)	3 (60)	5.2 (3.19)
	Biobrane alone	4	4.95 (3.91)	2 (50)	8 (5.23)
	Standard care	4	5.03 (2.5)	1 (25)	4.5 (0.58)
Guerid 2013 [29]	PC + k	15	46.9 (5.3)	5 (33.33)	NR
	Standard care	15	42.5 (3.1)	11 (73.33)	
Holmes 2018 [30]	Recall	83	39.5 (13.1)	70 (84.2)	10 (4.5)
	MSTSG				
Holmes 2019 [31]	Recall	30	39.1 (15.8)	25 (83)	2443 (1675)
	MSTSG				
Guo 2022 [32]	Hydrosurgery + RECELL	30			NR
	Surgical excision + dressing (standard care)	30			
Bairagi 2023 [33]	Recell/Biobrane	7	1.33 (0.92)	5 (71.4)	
	Biobrane alone	7	2.67 (1.84)	1 (14.3)	
	Silver dressing	8	1.75 (1.56)	4 (50)	
Iman 2013 [34]	Cell spray	18	28.5 (9.9)	8 (44)	1.44 (0.7)
	Cell injection	10	29.2 (4)	5 (50)	1.3 (0.67)

## Supplementary Materials

The following supporting information can be downloaded at https://www.mdpi.com/article/10.3390/medicina61030529/s1, Figure S1: Vancouver Scar Scale Value; Figure S2: Incidence of complete healing at 4th week; Figure S3: Infection Rates; Figure S4: Patients requiring another intervention.
