# Supplementary material for: Effect of Autologous Skin Cell Suspensions Versus Standard Treatment on Re-Epithelialization in Burn Injuries: A Meta-Analysis of RCTs
Source: Medicina (Kaunas). 2025 Mar 18;61(3):529. doi: 10.3390/medicina61030529 (PMC11943764; doi:10.3390/medicina61030529)
Supplement: Supplementary file 1 [file medicina-61-00529-s001.zip › medicina-3497193-supplementary.docx]

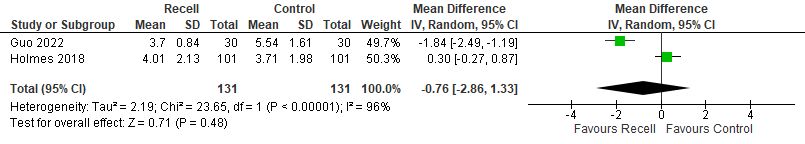

Figure S1 showing Vancouver Scar Scale Value

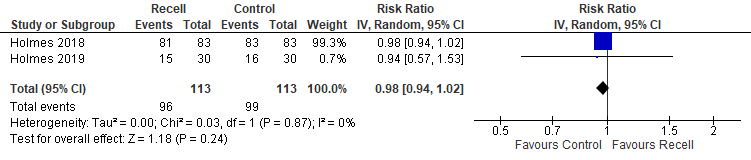

Figure S2 showing Incidence of complete healing at 4th week

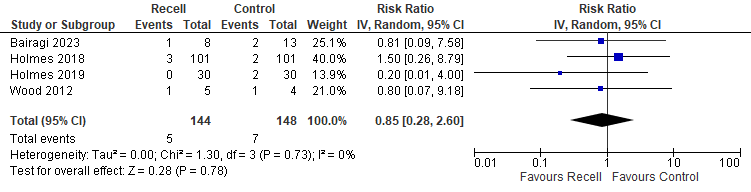

Figure S3 showing Infection Rates

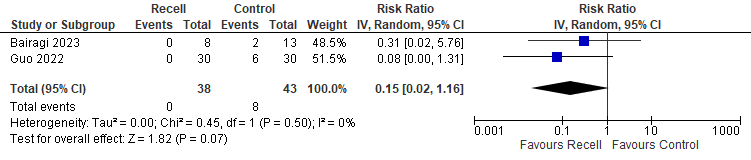

Figure S4 showing Patients requiring another intervention
